# Osteoimmunomodulatory effects of biomaterial modification strategies on macrophage polarization and bone regeneration

**DOI:** 10.1093/rb/rbaa006

**Published:** 2020-05-09

**Authors:** Yajuan Xie, Cheng Hu, Yi Feng, Danfeng Li, Tingting Ai, Yulei Huang, Xiaodan Chen, Lijia Huang, Jiali Tan

**Affiliations:** Guangdong Provincial Key Laboratory of Stomatology, Department of Orthodontics, Guanghua School of Stomatology, Hospital of Stomatology, Sun Yat-sen University, Guangzhou 510055, P. R. China

**Keywords:** biomaterials, modification, osteoimmunomodulation, macrophage polarization, osteoimmune environment, bone regeneration

## Abstract

Biomaterials as bone substitutes are always considered as foreign bodies that can trigger host immune responses. Traditional designing principles have been always aimed at minimizing the immune reactions by fabricating inert biomaterials. However, clinical evidence revealed that those methods still have limitations and many of which were only feasible in the laboratory. Currently, osteoimmunology, the very pioneering concept is drawing more and more attention—it does not simply regard the immune response as an obstacle during bone healing but emphasizes the intimate relationship of the immune and skeletal system, which includes diverse cells, cytokines, and signaling pathways. Properties of biomaterials like topography, wettability, surface charge, the release of cytokines, mediators, ions and other bioactive molecules can impose effects on immune responses to interfere with the skeletal system. Based on the bone formation mechanisms, the designing methods of the biomaterials change from immune evasive to immune reprogramming. Here, we discuss the osteoimmunomodulatory effects of the new modification strategies—adjusting properties of bone biomaterials to induce a favorable osteoimmune environment. Such strategies showed potential to benefit the development of bone materials and lay a solid foundation for the future clinical application.

## Introduction

Millions of people worldwide suffer from bone defects. Bone healing is affected especially for the individuals with systemic diseases like osteoporosis, osteoarthritis, and diabetes [1]. Bone grafting is an essential method of bone loss treating. However, owing to the invasive ways, functional defect of the donor site and the limited satisfying grafts, it is not an ideal choice for a considerable proportion of patients [2]. From this perspective, developing more practicable bone substitute has a promising prospect. In recent decades, biomaterials as potential bone substitute have developed rapidly and been in great demand to work as the alternative therapeutic tools of bone regeneration [3]. However, biomaterials have always been considered as the foreign bodies to result in foreign body reaction (FBR) along with excessive inflammation, tissue destruction, and fibrotic encapsulation, interfering with the speed and quality of osseointegration [4].

Numerous traditional design theories focus on the composition formation and structure fabrication to obtain inert biomaterials, expecting to minimize the immune actions caused by biomaterials [5]. Although progress has been made in the field of bone tissue engineering, various kinds of bone biomaterials fabricated in the traditional ways did not work functionally in practical treatment and many were only feasible in laboratories, which indicates the traditional design principles may be inadequate [6]. Clinical innovation and practical application are hindered, due to the lack of profound understanding of the biological processes during bone remodeling.

Currently, allowing specific cell responses has been reported to contribute to successful implantation. According to the mechanism of bone biology, bone formation is actualized via the close cross-talk of multiple systems. Among them, immune system deserves a place [7]. Events in the bone remodeling process like structural support, hematopoiesis and mineralization all need the close biological cooperation of skeletal system and immune system and they share various kinds of cells, cytokines, and signaling pathways [8]. The surprising intimate connection between the two systems is emphasized and defined as ‘osteoimmunology’ [9].

Nowadays, increasing strategies have confirmed the important position of immune reaction in the interactions. It inspires the shift from ‘immune-evasive’ biomaterials to ‘re-programming’ ones [10]. Osteoimmunomodulatory strategies aim to enable the biomaterials to modulate local immune environment from pro-inflammatory to be in favor of healing and regeneration [11]. This review outlines the intimate connection between the skeletal and immune system and highlights how can we integrate these underlying mechanisms into biomaterials modification. These strategies will provide ideas for new effective bone substitute development to become the potential solution to the challenges in osseointegration and osteogenesis.

## Interplay among biomaterials, immune system and skeletal system

### Immune response induced by biomaterials

**Figure 1.**
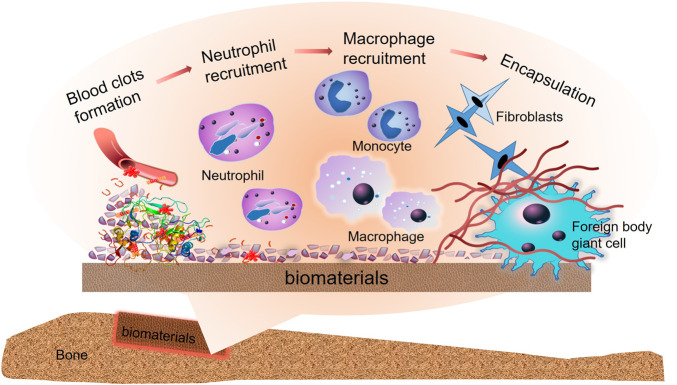
Biomaterials elicit immune reactions. As the implantation proceeds, the blood clots consist of protein, growth factors, cytokines, and MMPs adsorbed on the biomaterials surface and the injured area, which trigger a series of reactions in immune system. The neutrophils are recruited, and then monocytes gather and differentiate into activated macrophages which lead the secretion of various cytokines and take up biomaterials as foreign bodies by forming a fibrin matrix around the biomaterials

The host response is stimulated by biomaterials. Adsorption of proteins, recruitment of immune cells and secretion of signaling molecules are necessary for the healing process [12]. As the implantation proceeds, the protein layer and complement factors rapidly adsorb on the surface of biomaterials to form blood clots rich in growth factors, cytokines and matrix metalloproteinases (MMPs), thus to promote immune response and recruit neutrophils [13]. Activated platelets regulate the migration of monocytes which then differentiate into macrophages. The macrophages adhere and then form a fibrin matrix around the biomaterial, to regard the biomaterial as foreign body and try to phagocytosis and remove it. When the biomaterial cannot be swallowed and removed, the inflammatory response delays. And, a fibrous envelope is formed onto the substance to isolate it from the surrounding tissue (Fig. 1). The FBRs and fiber wrapping around the implant can directly affect the efficacy and result in the failure of implantation [4]. Therefore, the fate of bone biomaterials, to a great degree, depends on the immune response.

### RANK/RANKL/OPG axis

Bone cells including osteoblasts and osteoclasts control bone formation and resorption, and the delicate regulations of them make much sense. Osteoblasts play a major role in osteogenesis; they derive from MSCs which secret bone-matrix proteins and promote mineralization [14]. As for osteoclasts, they have the same hematopoietic origin as the myeloid precursor cells which produce macrophages and myeloid dendritic cells (DCs) [15]. During the osteoblastogenesis, various factors, for example, Runx2, β-catenin, and NFAT are involved [16]. During the osteoclastogenesis process, ruffled membranes act as the isolated extracellular microenvironment to bridge matrix recognizing integrins and cytoskeleton. The demineralization of bone is achieved by the acidifying vesicles secreted by osteoclasts. Lysosomal protease and cathepsin K are responsible for the organic component [17].

**Figure 2.**
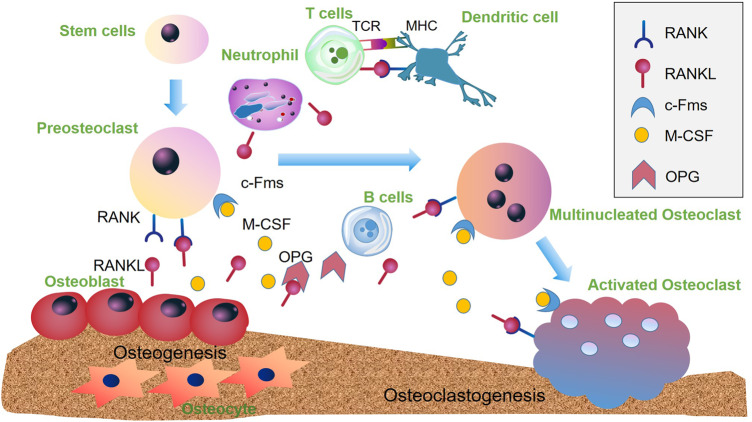
The function of RANK/RANKL/OPG pathway in osteoclastogenesis. Osteoblasts produce RANKL that is able to bind to the receptor RANK, leading osteoclast precursor cells to differentiate into preosteoclasts which can then fuze to nonfunctional multinucleated osteoclasts. After activation, the mature osteoclasts are formed to initiate bone absorption. OPG can also bind to RANKL to interfere with RANKL–RANK. M-CSF released from osteoblasts acts as a potent stimulator of RANK when binds to c-Fms. Neutrophils can also express RANKL to regulate osteoblast and trigger bone resorption. DCs are able to trans-differentiate into osteoclasts via RANK/RANKL pathway and interactions with CD4+ T cells

RANKL/RANK/OPG axis is the most crucial pathway in bone metabolic process (Fig. 2). Receptor activator of nuclear factor kappa-B ligand (RANKL) is a type II membrane-bound protein expressed on osteoblasts, bone marrow stromal cells (BMSC), T lymphocytes and neutrophils. As an important signal, it regulates osteoclast differentiation and participates in the physiologic and pathologic bone resorption. Besides, RANKL is able to stimulate DCs, playing an important role in osteoimmune environment [18]. Receptor activator of nuclear factor kappa-B (RANK) binds on the mature osteoclasts and their precursor; RANK is under the regulation of TNF receptor-associated factors 6 (TRAF6), which mediates gene expression of osteoclast survival and function. When RANKL interacts with RANK to form the RANKL/RANK system, the resorption events begin [19]. OPG (TNFRSF11B) is mainly released by osteoblasts. Be the decoy receptor for RANKL, OPG can compete with RANK and antagonize the effects of RANKL/RANK, to suppress the osteoclast differentiation and activation. So the ratio of RANKL/OPG is of much significance [20]. Macrophage colony-stimulating factor (M-CSF) is expressed by osteoblasts and binds to receptors (c-Fms) via the Akt and MAP kinase pathways. M-CSF can stimulate the expression of RANK, induce pre-osteoclast differentiation and regulate osteoclast apoptosis and survival. The physiological equilibrium of the RANK/RANKL/OPG pathway is pivotal in bone metastasis [21].

### Role of macrophage and the polarization

**Figure 3.**
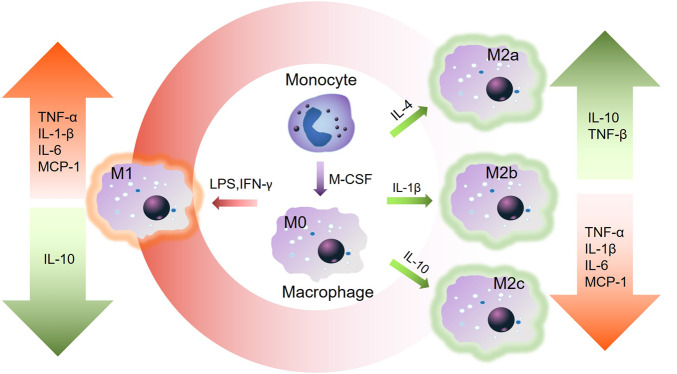
Polarization of macrophage and secretion of cytokines

Various kinds of immune cells get involved in regulating bone dynamics. As an important part of innate immunity, macrophages have aroused the most discussion [22]. And interestingly, macrophages are also pivotal in bone metabolism [23]. Indeed, given the proximity of macrophage lineage cells to bone cell, macrophages play a vital role. Bone marrow provides the same microenvironments for both bone and immune cells to harbor and share interrelated cellular and signaling pathways to engage and cooperate tightly in bone metabolism. It has been reported that macrophages can interact with mesenchymal precursors and promote osteoblast formation. To achieve this, various kinds of growth factors including oncostatin M are involved. In addition, the activation state and the polarization make sense. Currently, activated macrophages have been identified to potentially facilitate bone formation and accelerate tissue repair [24]. The enhanced function of alkaline phosphatase and increasing level of collagen I were observed, facilitating osteogenic differentiation, when the marrow stromal cells were co-cultured with monocyte/macrophage lineage [25]. Besides, osteoinductive factors including bone morphogenetic protein-2 (BMP-2) from monocyte/macrophage lineage have osteoinductive effects [26]. Macrophage has two major phenotypes named M1 and M2, respectively [27]. Previous studies have indicated that the balanced function of M1 and M2 is of much significance. Particularly, macrophage polarization states can switch, to adapt to the cytokine and microenvironment. And in turn, the different phenotypes have different effects [28]. M1 leads inflammatory response to clear the damaged tissue at the initial stage. M2 promotes the healing and regeneration process [29]. M1 can promote antigen presentation and Th1 differentiation, release IL-1β, TNF-α and IL-12 to up-regulate reactive oxygen species genes and promote pathogen killing and the activation of inflammation [30]. IFN-γ, LPS, and M-CSF are pro-inflammatory cytokines to stimulate M1 polarization [30]. Anti-inflammatory M2a and M2b initiate Th2 lymphocyte through IL-10, IL-1ra and IL-6. M2 phenotype is induced by various signals and secretes kinds of cytokines which promotes cell proliferation, differentiation and tissue repairing. Stimulated by IL-10, M2c can assist tissue remodeling and inhibit inflammation via releasing TGF-β and IL-10 [31]. These signals from the local environment benefit the vascularization of regenerative biomaterials. M2 works throughout the whole healing process thereby improving the integration of the biomaterials [32]. The ratio of M1 and M2 makes much sense. Consistently high M1 response, an extended period of M1 and the lack of M2 will cause severe FBR and chronic inflammation, prolonged immune response, delayed tissue healing and the failure of biomaterial integration [33]. The switch between the M1 and M2 phenotypes is regulated by diverse factors such as the cells, cytokines and miRNAs, and in turn, when the macrophage phenotype has been changed, their inflammatory cytokines secretion and the gene expression will also change, thus affecting the local immune environment [34]. The better understanding of immune cells, especially the proper control of cytokines releasing and M1–M2 polarization, will provide inspiration for the bone regeneration field (Fig. 3).

Other immune cells also play an important role. T cells have various subsets. Both Th1 and Th2 suppress osteoclast formation via the secretion of IFN-γ and IL-4. Th17 cells and Th17-related cytokines have been currently identified to significantly lead to higher level of RANKL, inducing osteoclastogenesis and involvement in bone diseases [35]. Th17 differentiation is triggered by TGF-β and IL-6 [36]. CD4+ T cells suppress osteoclast differentiation via IL-4, IL-10 and CTLA-4 [37], while NK T express M-CSF and RANKL to induce osteoclastogenesis [38]. OPG produced by B cells inhibits osteoclast function at the present of Th1 cytokines while playing a catalytic role via Th2 cytokines. B cells can cooperate with T cells to up-regulate OPG via CD40/CD40L co-stimulation pathways [39]. DCs affect T cell immune function. Mature DCs can activate Th17 cells and up-regulate IL-17 to promote osteoclastogenesis [40]. Additionally, DCs can trans-differentiate into osteoclasts via RANKL and M-CSF pathway, in the interactions with CD4+ T cells [41]. Mast cells also participate actively in osteoclastogenesis. The enhancement of systemic mastocytosis facilitates the bone loss. Additionally, the deficiency of histamine may contribute to decreasing bone loss [42]. Neutrophils can also express RANKL to regulate osteoblast and trigger bone resorption [43]. Considering the recruitment of neutrophils is closely connected with the adsorption density of proteins on biomaterials and then affects the biocompatibility, it is an essential cue to design biomaterials that regulate the neutrophils functions. MSCs and their secretions feature with immunoregulation [44]. For example, scaffold construction and composition may have an influence on the cell surface marker (like MHC-1, and MHC-2) [45] and immunoregulatory functions [46].

### Diseases related to bone immunity

Maintaining normal skeletal homeostasis needs to keep a dynamic balance of osteogenesis and osteoclastogenesis, which relies on the delicate regulation. Abnormal functions of the immune responses at the site of injury and throughout the body may cause degenerative diseases like rheumatoid arthritis (RA), osteoporosis and periodontal disease (PD), causing bone loss [47]. RA is a common autoimmune diseases with abnormal immune function which interferes with the bone homeostasis in the synovial joint, increasing the fracture risk. Cells include osteoclasts, T cells, monocytes and synovial fibroblasts all participate in the process and are mainly regulated by the above-mentioned RANKL signaling system and various kinds of cytokines [48]. Osteoporosis is common in postmenopausal women, because the low level of the estrogen can result in the bone loss. Bone cells including osteoclasts, osteoblasts, and osteocytes; immune cells including lymphocytes, macrophages, and DCs express the nuclear estrogen receptor ERα; So the inter-connectivity of the estrogen, bone cells and immune cells have intimate relationship and multiple effects of each other [7]. As for PD, bacterially derived factors and antigens activate the innate immune system. Cytokine including IL-1 and TNF-α, cells including B and T cells mainly participate in the pathology. And, via the RANK/RANK/OPG axis, the osteoclastogenesis and subsequent bone loss are initiated [49]. Early in the bone remodeling, acute inflammation triggers the recruitment T-cells and monocytes, leading the secretion of TNF and IL-6 that facilitate MSCs to differentiate into osteoblasts [50]. However, if the infection goes into long-term inflammation, the existing pro-inflammatory cytokines may negatively affect bone regeneration. Accumulating evidence indicated that, to treat these diseases, it was necessary to reduce osteoclast activity while to facilitate bone formation [51].

With the increasing comprehension, it has been discovered that the delicate cooperation of immune and skeletal systems and complex interplay of cells, signaling molecular and pathways in osteoblastogenesis and osteoclastogenesis are of much significance for bone healing [52].

## Osteoimmunomodulatory effects of modification strategies

**Figure 4.**
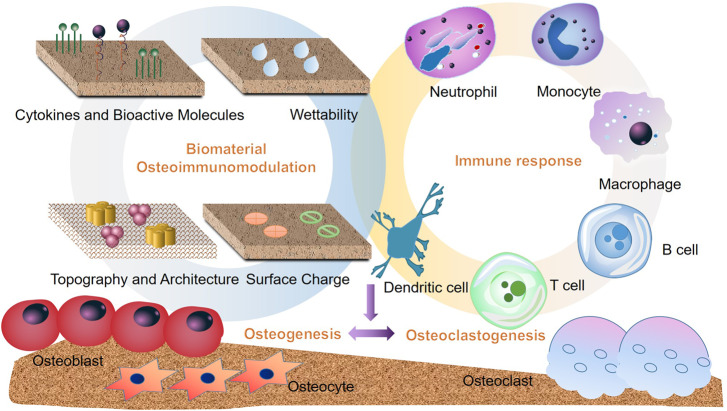
Modification strategies such as topography, wettability, surface charge, cytokines and bioactive molecules release of bone biomaterials can modulate the osteoimmune environment

Biomaterials can be modified to perform ‘osteoimmunomodulatory’ ability. Osteoimmunomodulatory biomaterials regulate the cell behaviors and osteoimmune environment systematically, thereby affecting the bone regeneration. Currently, incorporation of bioactive molecules or passive intervention of the physical and chemical characteristics to regulate appropriate immune response are considered as the main strategies [53]. Here, we summarized some osteoimmunomodulatory strategies of bone biomaterials (Fig. 4).

### Biomaterials applied in tissue engineering

**Figure 5.**
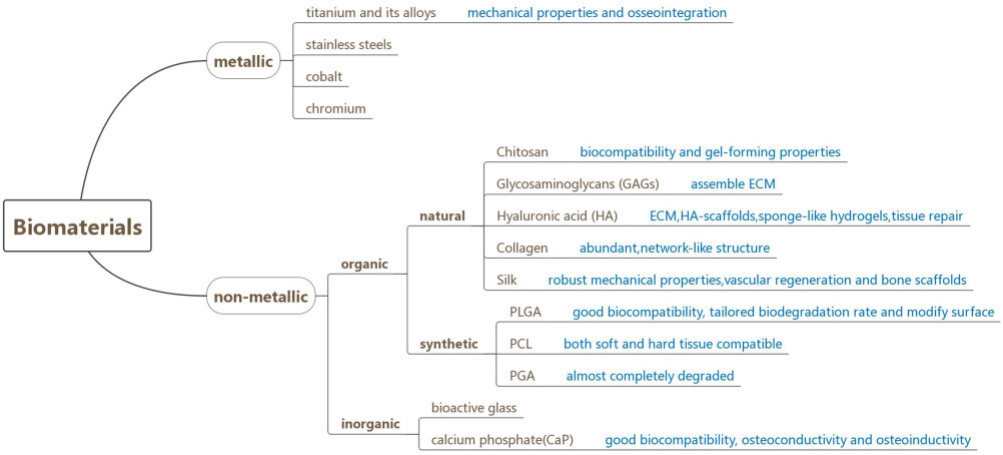
Biomaterials which are mainly applied in tissue engineering and their characteristics

Biomaterials are usually classified into metallic materials and nonmetallic materials. Several kinds of biomaterials applied widely in regenerative medicine and tissue engineering are summarized in Fig. 5. Metal materials including stainless steels, cobalt, chromium, titanium and their alloys are commonly applied in orthopedics and dentistry [54]. Among them, titanium is most broadly employed in oral, maxillofacial and craniofacial surgery for bone repair due to its outstanding mechanical and osseointegration properties which help bones to be attached directly to the metal surface with less fibrous tissue formation [55]. Nonmetallic materials are divided into the organic and the inorganic. Organic materials include natural polymers and synthetic polymers. Polymeric materials occupy a large proportion of the tissue engineering field [56]. As for natural polymers, the key compositions are polysaccharides and proteins. The resemblance to the native extracellular matrix (ECM) allows them to be potentially applied to build highly biocompatible scaffolds [57]. For instance, chitosan is derived from chitin, characteristic with splendid biodegradability, biocompatibility, immunogenic and gel-forming properties [58]. Glycosaminoglycans (GAGs) participate in signaling of cells and assembling of the ECM [59]. Hyaluronic acid (HA) as the main composition of ECM is able to regulate tissue injury and accelerate tissue repair [60]. Adopting HA to fabricate scaffolds has been extensively researched [61]. Collagen is abundant and its natural network-like structure is beneficial to the formation of highly organized, 3D bone substitute [62]. With good biocompatibility, mechanical properties and biodegradability, silk has been extensively discussed [63]. Furthermore, silk is able to form hydrogel to obtain hydrophilic properties. And, silk fibroin-based materials are applied for vascular regeneration and bone scaffolds [64]. Natural polymers do not induce a typical foreign body response with decellularized tissue [65]; they are easily degraded by enzymes and release immunomodulatory molecules [66]. As tissue engineering scaffolds, hydrogels are similar to the ECM structure, maintaining the natural biochemical signals and delivering biological molecules [67]. HA and collagen are both biocompatible polymers and the composite hydrogels are not easily to be repelled by host, which is suitable for the implantation into human body [68]. Currently, synthetic polymers have a bright biotechnological application prospect [69]. With good biodegradability and surface modification potential, poly(lactic-co-glycolic acid) is widely used in the field of biomedicine [70]. Poly(caprolactone) (PCL) can be constructed in films, fibers and microparticles and has high bone inductive potentiality [71]. Poly(glycolic acid) is a bone-friendly tissue engineering biomaterial that can be almost completely degraded *in vivo* conditions within months [72]. However, the synthetic materials are prone to trigger classic FBR and can be degraded during implantation [73]. Thus, it is necessary to undertake prophylactic drug treatments on the surfaces of synthetic polymers to avoid potential complement-mediated reactions. To obtain better scaffold performance and make the natural polymer materials moderately operable, developing the customized immune regulatory structure of synthetic polymer materials is widely accepted [74]. As for inorganic materials, they mainly include bioactive glass and calcium phosphate, which are characterized by the good biocompatibility, osteoconductivity and osteoinductivity, owing to the similar chemical and structural formulations to bone tissue [75, 76]. Currently, calcium phosphate materials of various compositions and crystal phases have been developed and utilized in bone regeneration [77]. Composite materials are composed of two or more types of materials and the combination of several kinds of components can reinforce complementary advantages and obtain better performance than a single component [78].

### Osteoimmunomodulation by delivering cytokines and bioactive molecules

There are various kinds of signal factors and cytokines in the osteoimmune environment. Their interactions and functions are complicated and some data even indicate their dual roles. Regulation of the signal factors and cytokines on osteogenesis and osteoclastogenesis will contribute to carrying out immunomodulatory approaches.

Some cytokines showed anti-osteoclastogenic functions, and modulation of the temporal and spatial pattern to coordinate the different stages of the bone remodeling process have been investigated [79]. Kara *et al*. have designed scaffolds with sequential IFN-g and IL-4 release profiles in the murine subcutaneous implantation model to enhance vascularization. On the scaffolds, IFN-g was shortly released to facilitate M1 polarization. Then IL-4 was released to induce M2 polarization. The outcomes indicated that the osteoimmunomodulated scaffolds regulated angiogenic behaviors. And, via modulating macrophage polarization, the immune microenvironment of cells and cytokine secretion profiles can be modulated [80]. Besides, IL-4 [81] and IL-33 are also inhibitors of osteoclast function [82]. IL-33 accompanied by IL-4 was able to promote mononuclear to differentiate into DCs and macrophages, down-regulate osteoclast precursors and osteoclast differentiation to interfere with osteoclastogenesis [83]. Likewise, Spiller *et al.* designed scaffolds with sequential cytokines release. The results indicated that VEGF was released by M1 to initiate the angiogenesis. PDGF-BB and MMP-9 were secreted by M2 to regulate vessel maturation. The different function of M1 and M2 is crucial in the angiogenesis and scaffold vascularization [84]. Other cytokines like IL-10, IL-12, IFN family also play an important role in bone regeneration via different pathways. IL-10 can suppress bone resorption by interfering with the expression of nuclear factor of activated T-cells cytoplasmic 1, an important factor for osteoclast differentiation [85]. IFN family mainly includes IFN-α, IFN-β and IFN-γ that all can inhibit the differentiation of osteoclasts. IFN-α and IFN-β participate in the innate immune responses and IFN-γ stimulates macrophage [86]. IFN-γ suppresses osteoclast differentiation via a negative feedback loop for RANK/RANKL/OPG pathway [87].

On the contrary, there are other cytokines like TNF-α, IL-1 and IL-6 that related to excessive inflammation with higher RANKL/OPG ratio and more active osteoclast functions. Inhibiting these inflammatory cytokines has been demonstrated to be a powerful method to reduce inflammation and inhibit osteoclastogenesis. TNF can regulate osteoclast precursor cells via enhancing c-Fms, promoting osteoclast differentiation. TNF-α is prone to lead nonunion fractures and delay fracture healing, which is consistent in RA [88]. TNF-α has also been described to inhibit osteoblast differentiation as well as collagen formation via the down-regulation of IGF-1, Osx, and Runx2. Furthermore, TNF-α induced osteoblasts apoptosis via FaseFas ligand signaling [89]. However, previous researches have also revealed the concentration dependence, time dependence and dual function of TNF-α on bone metabolism. TNF-α of low concentration has performed the opposite functions in murine slow-healing fractures model, via enhancing Runx2, Osx, osteocalcin, ALP and BMP-2 to facilitate osteogenesis and fracture healing, which indicates the precise modulation of TNF-α makes sense for bone regeneration [90]. IL-1 (IL-1α and IL-1β) promotes bone resorption via the direct regulation of osteoclasts and enhancing OPG and RANKL expression [91, 92]. In addition, IL-1 inhibits the osteogenic-differentiation of MSC via interfering with Wnt signaling and suppresses osteogenesis through NF-κB and MAPK signaling [93]. IL-6 is identified to participate the excessive bone loss via participating in the RANKL expression and the osteoclast formation [94]. IL-17 leads to local inflammation, RANKL expression and activates osteoclast precursors by inducing TNF-α and IL-1 [95]. Chang *et al.* found pro-inflammatory TNF and IL-17 could interfere with osteogenic differentiation of MSCs and stimulate IκB kinase (IKK)-NF-κB. It has been observed that inflammation response was reduced and bone MSC-mediated formation was promoted by delivering IKKVI (IKK small-molecule inhibitor) [96]. Apart from the above-mentioned cytokines, IL-11 [97], IL-8 [82], CXCL12 and CCL2 may also be involved in the osteoclastogenesis [98].

Bone morphogenetic protein-2 is frequently used for osteogenesis. Wei *et al.* incorporated 20 mg/ml BMP-2 into the gelatin sponge to study the immunoregulatory role on macrophage and osteogenesis. And, increased macrophage recruitment has been observed. Furthermore, IL-1β, IL-6, and inducible nitric oxide synthase (iNOS, M1 marker) reduced, implying the positive osteoimmunoregulatory role of BMP-2 under inflammatory conditions. Additionally, BMP-2 has been described to activate macrophage alone via pSmad1/5/8 signaling pathway and produce a positive feedback loop via enhancing angiogenic factors. Conditioned medium collected from BMP-2-stimulated macrophage facilitated the osteogenic differentiation of BMSC, suggesting that BMP-2-induced osteogenesis could be involved in the meditation of the local osteoimmune environment [99]. In another study, considering that BMP-2 performs an osteoinductive role to initiate the osteoblastic differentiation and OPG as a RANKL inhibitor to block osteoclastic function, Bougioukli *et al.* [100] combined BMP-2 with OPG. By comparing the BMP-2 group, the outcomes suggested that osteoclasts can hinder the healing function of BMP-2 and using OPG to inhibit RANKL could facilitate BMP-2 efficacy. In OA cartilage, RANKL/OPG ratio was higher [101]; suppression of RANKL via systemic or intra-articular application of OPG may prevent bone and cartilage degradation [102].

Specialized pro-resolving mediators are endogenous molecules that suppress inflammatory cells recruitment and interfere with macrophage phagocytosis while promoting M2 macrophage polarization. The utilization of pro-resolution mediators facilitated to alter the inflammation [103]. Vasconcelos *et al.* used the pro-resolution lipid mediators Lipoxin A4 (LxA4) and Resolvin D1 (RvD1) to regulate chitosan scaffolds. Reduced inflammatory IL-1, IL-16 and TNF-α, less fibrous capsule, more CD206+ cells and down-regulated CCR7+ cells were observed [104]. In another study, Yin *et al.* [105] fabricated biomimetic anti-inflammatory nano-capsule which was coated with cytokines receptors on the surface to block activated pro-inflammation and coated with Resolvin D1 inside to facilitate M2 macrophage polarization. Besides, delivering drugs were also studied. For example, Riccitiello *et al.* fabricated electrospun nanofibers to deliver and release drug RSV which was able to down-regulate RANKL-mediated osteoclastogenesis and prohibit the maturation of osteoclast precursors [106]. These modification strategies served as promising potentials to be applied in bone regeneration. There were also strategies which introduce inorganic ions to modulate biomaterials. Shi *et al.* prepared hypoxia-inducing copper-doped mesoporous silica nanospheres (Cu-MSNs) with sustained released Si and Cu ions. The hypoxia induced by Cu was reported to promote angiogenesis. And, Cu-MSNs showed the osteoimmunomodulatory properties to enhance osteogenesis by stimulating OSM pathway, which suggested the potential for bone tissue therapies [107].

In general, based on the bone metabolism mechanisms, different approaches have been investigated and it has been the current development trend to design bone materials by delivering osteoimmunomodulatory cytokines and bioactive molecules (such as drugs, mediators, ions) to regulate the macrophage polarization and RANK/RANKL signaling pathways, to directly or indirectly modulate the osteogenesis. Although these discoveries are helpful to achieve a favorable osteoimmune environment, many exact mechanisms are still complex and call for further investigations.

### Osteoimmunomodulation by surface topography and architecture

Potential of biomaterials to regulate the function of immune cells has been reported, which encouraged the design of biomaterials to trigger an appropriate immune response at the site of implantation. Surface modification of biomaterials has drawn more and more attention, and the osteoimmunomodulatory properties can stimulate immune cells functions, making it possible to build a favorable osteoimmune environment for bone regeneration [108]. The polarization ability of macrophage is quite sensitive to the physicochemical properties of biomaterials. Immunomodulatory regulations of macrophage and the phenotypes may be potential good strategies to meditate the local environment for bone tissue engineering [109].

Surface roughness is an important modification method to regulate osteoblastogenesis and osteoclastogenesis, which was heatedly discussed. There are various approaches to modify surface roughness such as polishing and sandblasting. It has been evidenced that micropatterned scaffold can positively modulate the osteoimmune environment and the modified implant surface was considered able to improve the implantation success rate. For instance, Hotchkiss *et al.* described that Ti surfaces with microroughness benefited the healing process. Because while smooth Ti facilitated M1 polarization with increased pro-inflammatory IL-1β, IL-6, and TNF-α, the Ti surfaces modified by microroughness promoted M2 with increased anti-inflammatory IL-4 and IL-10 [110]. Likely, in another study, Zhu *et al.* compared the smooth-surface BCP with hBCP (BCP with micro-whiskers and nanoparticles hybrid-structured surface). In hBCP group, it was observed that pro-inflammatory TNF-α and IL6 were prominently reduced. Meanwhile, the gene expressions of SLC20a1 and ATP2B2 were promoted, which were favor of phosphate and calcium ion transporters. And FGF23, known as a stimulator of osteogenic genes was >3-fold. It positively regulated osteogenic differentiation by facilitating Runx2, OCN and ALP. Additionally, it also hindered the expression of adipogenesis-related genes [77]. Additionally, the roughness of the surface not only influenced the cytokines secretion but also had an effect on angiogenesis and BMSC function. Yang *et al.* [111] demonstrated that rough titanium–blood interactions can promote the proliferation and recruitment of rat BMSC. And also, Pan *et al.* reported osteoimmunomodulatory potential of the hierarchical macropore/nanosurface. Enhancing function of CD206, Arg1 (M2 marker), anti-inflammatory IL-4, IL-10 and IL-1ra was observed. Up-regulated osteogenic differentiation, BMP-2 secretion, and angiogenesis of human umbilical vein endothelial cells were also described. While CD11c (M1 marker), pro-inflammatory iNOS, IL-1β, IL-6, TNF-α and IFN-γ were down-regulated. The mechanism may be the multi-directional structure that regulated the cytoskeleton tension [112].

TiO2 nanotube structures were reported to provide more reactive area for the protein aggregation, interactions and macrophage adhesion, owing to their empty pore spaces and gaps between nanotubes. For instance, fibronectin and vitronectin acted as ligands absorbed on the surfaces to interact with macrophage through RGD-integrin receptor domains, followed by the secretion of cytokine [106]. Lu *et al.* [113] compared the different effects of the different diameters on TiO2 nanotubes, indicating that 80 nm group offered more appropriate sites that preferentially adsorb fibronectin and vitronectin to regulate macrophage adhesion and proliferation, while inhibiting IL-1β, IL-6, TNF-α, MCP-1 and MIP-1α expression.

Electrospun fibers have been recently regarded as promising bone regeneration scaffolds, because the morphology is significantly similar to the natural collagen fibrils [114]. Saino *et al.* made a comparison the effects of fibrous poly (l-lactic) (PLLA) scaffold with different fiber diameter and alignment. The results demonstrated that fiber diameter influenced the secretion of pro-inflammatory cytokines. Nanofibrous PLLA group showed much less inflammation than films and microfibrous ones and PLLA films showed the most infiltration of foreign body giant cells [115].

Pores are important for vascularization and nutrients transport; porous materials are wildly applied in the fabrication of engineering structures [116]. Pore size and porosity have been reported to act as important regulatory cues. Garg *et al.* reported that the increase of pore diameter and higher porosity of polydioxanone scaffold enhanced Arg1 (M2 marker) and angiogenic VEGF, TGF-β1, and bFGF. Additionally, it down-regulated iNOS, while materials with smaller pores or random size facilitate M1. It has been identified by microscopic analysis that large pores allowed more Mφ to orient in the 3D space while smaller pores were difficult to infiltrate Mφ [117]. Furthermore, a balance between the scaffold porosity and the structural robustness is supposed to be taken into account to ensure the strength, which is quite essential for the scaffold. For one thing, increasing structural porosity may affect the function of macrophage and the regeneration environment. For another, it may have a negative impact on mechanical strength.

Regulating the surface properties or structure construction of biomaterials can change the protein layer and regulate different receptor binding as well as signal transduction, to lead changes in cell response, for example, to modulate the adhesion, activation and fusion of macrophage and FBGCs. The cellular response is usually triggered by the adsorbed factors, not the surface itself. And, various kinds of cytokines are involved in the regulation process [109]. In this regard, the modification of the physical of biomaterials can regulate the activation of immune cells especially on macrophages. Therefore, what has been found about the osteoimmunomodulatory behaviors of biomaterials on cellular behaviors provides strategies to optimally design the surface of bone biomaterials to regulate the osteoimmune environment. However, many mechanisms of different osteoimmunomodulation methods to decipher the complex interplay underlying cell behaviors still need further investigation.

### Osteoimmunomodulation by wettability

It is demonstrated that the osseointegration enhanced with the hydrophilicity increased. Hamlet *et al.* compared the different effects of hydrophilic-modified SLA (modSLA) and SLA Ti. The modSLA group promoted the expression of CD163 protein and Arg1 gene, marking the M2 polarization. And, the gene expression of TGF-β/BMP signaling pathway in osteogenesis was up-regulated. On the contrary, macrophage of the SLA group was prone to switch to an inflammatory phenotype and enhance the pro-inflammatory cytokine secretion; the osteogenic gene expression was not observed. It has been identified that the polarization of macrophages is under the influence of the signaling factors from the biomaterial surface. And, the hydrophilic micro-rough ones stimulated anti-inflammation polarization [118]. Likewise, in another study, murine macrophage cultured on the hydrophilic surface down-regulated the pro-inflammatory secretion of TNF-α, IL-1 and CCL2. Lower expression of the corresponding protein confirmed the change of the cytokine gene expression [119]. Furthermore, compared with the surface roughness modification, increased wettability showed more activation of the anti-inflammatory macrophage. Additionally, the hydrophilic micro-rough surface was showed to lead to higher level of anti-inflammatory cytokine secretion than the smooth one. What is more, increasing surface roughness and hydrophilicity leveraged a synergistic solution to promote osseointegration and osteogenesis. The mechanism was reported that the micro-rough surface allowed less contamination while enhanced surface energy, which could improve wettability [110]. Furthermore, functional groups like amino (–NH2), hydroxyl (–OH), carboxyl (–COOH) and hydrophilic molecules like polyethylene oxide (PEO) and polyethylene glycol (PEG) were commonly researched [120]. Bartneck *et al.* found that –COOH-modified nanoparticles were able to lead inflammatory M1 phenotype polarization and pro-inflammatory IL-1β, IL-6, TNF-α and CCL2. Nanorods with –OH was observed to enhance IL-1 and CCL2. While –NH2 termination nanorods resulted in anti-inflammatory M2. Although the immunomodulatory effect on macrophage phenotype was observed, the exact mechanism of the superior performance has not been clearly clarified [121].

The wettability of biomaterial is closely associated with the surface proteins, blood clot and fibrin formation. The surface protein layer can interact with immune cells and activate various factors as well as signal pathways. Especially, macrophages cultured on surface with different wettability have been found to change the protein expression profiles, thereby changing cytokine secretions. Hydrophilic biomaterials are usually observed protein resistance while hydrophobic biomaterials have inherent immunogenicity. What has been discovered above gives clues for modifying biomaterials characteristics to guide the desired cellular biological behavior.

### Osteoimmunomodulation by surface charge

Surface charge of biomaterials has also been reported to influence osteoimmune environment [122]. Brodbeck *et al.* compared the biomaterials with anionic functional group of poly (acrylic acid) and cationic functional group of poly (dimethylamino propyl acrylamide). The anionic substrate group increased IL-10 and decreased IL-8, while the cationic group down-regulated the important cytokine in the maturation of osteoblasts like IL-10 and IL-1RA. The outcomes showed that anionic group positively affected osteoblast function, while the cationic was prone to up-regulate pro-inflammatory secretion and interfere with osteoblasts activation [123]. Moreover, divalent cations may polarize macrophages. With Ca^2+^ and Sr^2+^ used to modify Ti surface, the enhancement of Arg1, MR, CD163 (M2 markers), TGF-β1, PDGF-B and VEGF were observed. BMP-2 released by macrophage was found to facilitate MSCs osteogenic differentiation and promote osteogenesis [124]. These results identified the up-regulated migration and proliferation of endothelial cells to enhance angiogenesis. Additionally, they also acted as the chemoattractant to circulate MSCs to induce bone regeneration, which indicates that the divalent cationic surface was prone to enhance osteogenic differentiation and osteogenesis [125]. Likewise, Bose *et al.* [126] demonstrated that Mg^2+^ and Si^4+^ in the TCP played a positive role in both osteogenesis and angiogenesis. Cell adhesion played a key role in cells differentiation. A positively charged culture surface enlarged the adhesion area of rBMMSC and promoted differentiation. However these results which described the early differentiation of rBMMSCs into osteoblast-like cells were not sufficient enough to explain the osteogenic differentiation of rBMMSCs [122]. What is more, the surface charge could be affected by other properties like chemical groups or roughness. So it would be difficult to carry out single-factor study of surface charge characteristics without any interference from other factors. Further future work could focus on the complex relationships between surface charges and cellular responses.

### Osteoimmunomodulation by decellularized ECM

Biomaterials and host immune system components interact directly in the presence of ECM, which is the noncellular milieu to build fundamental physical framework and deliver cells as well as biological factors. The function of extracellular environment cannot be ignored [127]. The matrix of ECM can store signal molecules, take part in cell–matrix interactions, activate enzymes and regulate cytokine to control cellular morphogenesis, differentiation, and homeostasis in biomaterial integration and tissue healing process [128]. Currently, the employment of decellularized ECM for the regulation of osteoimmune environment is promising. While the native functional groups are exposed or masked via the matrix isolation process, the bioactivity of the ECM can be changed. The ECM decellularization retains only the native structure and removes tissue immunogenic components to reduce antigen, so as to supports infiltration of the host cells and promotes tissue recovery [129, 130].

ECM components are of much significance. Scaffold embedded in GAG modified by chondroitin sulfate and heparan sulfate to obtain the sulfated GAG linked to serine-rich protein-forming proteoglycan; The polysaccharide matrix acts as a repository as well as regulators for signal molecules [131]. Cells are attached to ECM through a variety of surface receptors such as integrin and selectin, which can specifically adhere to adhesion domains of scaffolds or bind to the polysaccharide matrix to trigger signaling pathways [132]. For example, the negatively charged sulfate groups of proteoglycan can form electrostatic interactions with the positively charged surfaces of signal molecules, affecting the concentration, biological behaviors and stability of the signaling factors [129]. Growth factors are sensitive to proteolytic degradation. The combination with ECM allows the growth factors to interact with its specific ligands and prevents them from enzymatic cleavage and diffusion. Therefore, it is easier to satisfy the local concentration of growth factors needed for signal transmission [133]. In one study, adding high-sulfated hyaluronan down-regulated pro-inflammatory IL-1β, IL-6, IL-8, IL-12 and TNF-α while enhanced anti-inflammatory IL-10 and CD163 [134]. Huleihel *et al.* reported that matrix-bound nanovesicles to fabricate ECM bio-scaffolds, their miRNA cargo appeared to actively participate in the macrophage polarization. And, matrix-bound nanovesicles isolated from small-intestinal submucosa (SIS) and urinary bladder matrix (UBM) were found rich in miRNA1-5p, 143-3p and 145-5p. Inhibiting these miRNA had involvement with pro-inflammatory phenotype [135]. ECM glycoproteins have attracted much attention owing to the osteogenic potential and involvement in immune phenotype. S100A8 and S100A9 were damage-related molecular model proteins, which not only activated the osteogenic gene program but also had immunomodulatory function by interacting with the receptors of advanced glycation end products. Cartilage oligomeric matrix proteins and matrilin down-regulated the M1 polarization [136].

Furthermore, the type of immune response highly depends on the source of ECM, for different decellularization procedures result in different effects on macrophages. Dziki *et al.* demonstrated that M2 macrophage phenotype was observed with pro-remodeling and anti-inflammatory performance, when exposed to SIS, UBM, brain ECM (bECM), esophageal ECM (eECM), and colonic ECM (coECM). On the contrary, dermal ECM led to M1 phenotype, pro-inflammatory performance (iNOS+/Fizz1−/CD206−), while no significant difference has been observed between liver ECM (LECM) and skeletal muscle ECM (mECM) [137].

ECM architecture may also modulate macrophage phenotype polarization via micropatterning to modulate macrophage shape. McWhorter *et al.* demonstrated that elongation itself enhanced M2 markers and down-regulated pro-inflammatory cytokines without exogenous cytokines. Besides, elongation up-lifts M2-inducing IL-4 and IL-13 while avoiding M1-inducing LPS and IFN-γ. Furthermore, when the contractility forces of actin and actin/myosin were suppressed by inhibitors, shape-induced polarization is abrogated, which implies the importance of the cytoskeleton in modulating macrophage polarization via cell geometry. And, shape-induced polarization may act differently on cytokine-induced one via dissimilar pathway [138]. Mimicking the function of ECM contributes to obtaining optimal bone scaffolds that applied to tissue engineering.

The osteoimmunomodulatory effects of different modification strategies are listed as follows (Table 1).

**Table 1.** Modification strategies of biomaterials showed osteoimmunomodulatory effects

Properties	Modification strategies	Effects	References
Surface topography and architecture	TiO_2_ nanotube (80 nm)	↑ Macrophage adhesion and proliferation	[113]
		↓ Protein and TNF-α, MCP-1, IL-1β and IL-6	
	Hierarchical macropore/nanosurface	M2 polarization	[112]
		↑Anti-inflammatory genes expression	
		↑Osteogenic differentiation and angiogenesis	
		↓ Inflammatory genes expression	
	Micro-whiskers and nanoparticles hybrid- structured (hBCP)	↑ Collagen content	[77]
		↓ Inflammatory genes expression	
	Nanofibrous PLLA scaffolds	↓ Inflammatory response	[115]
	Ti surfaces with microroughness	M2 polarization	[110]
		↑ IL-4 and IL-10	
	Larger diameter fibers with larger pore sizes and porosity	M2 polarization	[117]
		↑ Angiogenesis with VEGF, TGF-β1 and bFGF	
		↓ Inflammation and the M1 marker iNOS	
		↑Mφ infiltration	
Wettability			
	Hydrophilic-modified SLA	↑ CD163 protein and Arg1	[118]
		↑ The TGF-β/BMP signaling pathway	
	Combination of increased surface roughness and hydrophilicity	↑ Bone healing and increases osseointegration	[110]
Surface charge	Anionic and cationic functional groups	Anionic surfaces ↑ osteoblast function	[123]
		Cationic substrate ↑ pro-inflammatory cytokines	
		Cationic substrate ↓ activation of osteoblasts	
	Ti implant with Ca^2+^ and ^Sr2+^	Divalent cationic surface ↑ osteoblasts effects	[124]
	Mg2+ and Si4+ in the TCP scaffolds	↑ Osteogenesis and angiogenesis	[126]
Cytokines and bioactive molecules	Sequential delivery of IFNg and IL4	M2 polarization	[139]
		↑ Vascularization	
	Delivering IKKVI	↑ MSC-mediated bone formation	[96]
		↓ Inflammation	
	Gelatin sponge incorporated with 20 mg/ml BMP-2	↑ Macrophage recruitment	[99]
		↓ M1 markers	
	Lipoxin A4 (LxA4), Resolvin D1 (RvD1)	M2 polarization	[104, 105]
		↓Neutrophils recruitment, pro-inflammatory cytokines and fibrous capsule	
	Copper-doped mesoporous silica nanospheres	↑ Osteogenesis and angiogenesis	[107]
	poly(ε-caprolactone) PCL and poly(lactic) acid (PLA) loading resveratrol	↓ RANKL and maturation of osteoclast precursors	[140]
Decellularized ECM	ECM glycoproteins	Cartilage oligomeric matrix proteins and matrilin ↓ the M1 polarization	[136]
	The origin of ECM	SIS, UBM, bECM, eECM and coECM: pro-remodeling and anti-inflammatory	[137]
		Dermal ECM: M1 phenotype, pro-inflammatory performance (iNOS+/Fizz1−/CD206−)	
	Matrix-bound nanovesicles	↑ miRNA1-5p , 143-3p and 145-5p.	[135]
		↓ M1 phenotype	
	Modify ECM architecture	↑ M2 phenotype	[138]
		↓Inflammatory cytokines without exogenous cytokines	

## Conclusions and future perspectives

Biomaterials as bone substitutes have always been considered to trigger FBRs. Traditional designing methods were prone to fabricate biomaterials which can minimize the host immune response. However, the obtained biomaterials were not to the satisfaction for the clinical application. Not content with that, numerous researches have struggled to study the biological characteristics of immune and bone modeling. Currently, the concept of osteoimmunology was inspiring, which mainly emphasized the interaction among biomaterials, immune system and skeletal system. Immune response was not necessarily bad news, allowing specific reactions has shown to facilitate bone remodeling process. These reactions were under the delicate cooperation of cells (both bone cells and immune cells), cytokines and signaling pathways. Biomaterials would not simply act as foreign bodies, they can be modified to obtain different characteristics thus influence the protein adsorption and signaling factors binding. Numerous studies have shown that the modification strategies like drug delivery, topography, structure, wettability, surface charge have been evidenced to individually or synergistically regulate immune functions and bone metabolism. Reviewing the existing studies, we summarized the osteoimmunomodulatory effects of the different modification approaches, which described encouraging results and may develop functional biomaterials to be potentially applied in bone tissue engineering. And, we found that a considerable amount of studies had confirmed the significant role of the modulation of macrophages phenotypes, which has also been widely researched and applied in many modification strategies, however, many of the exact mechanism were still unclear and called for further investigations. Moreover, as we know, compared with macrophages, there were relatively less attention on other cells like DCs, B cells and T cells. Even though some studies mentioned their participation in the osteoimmune environment, very few studies could clarify their roles and potential in bone remodeling. Furthermore, the osteoimmunomodulation may also be a double-edged sword because the signaling pathways are complex and also play a dual role, it is difficult to control one element and other factors remain the same. Therefore, there is a need to carry out further studies on fabricating functional biomaterials to modulate the material-host response accurately and actively for bone regeneration.
